# Current strategies of mechanical stimulation for maturation of cardiac microtissues

**DOI:** 10.1007/s12551-021-00841-6

**Published:** 2021-09-10

**Authors:** Maria Carlos-Oliveira, Ferran Lozano-Juan, Paola Occhetta, Roberta Visone, Marco Rasponi

**Affiliations:** 1 Department of Electronics, Information and Bioengineering, Politecnico di Milano, Via Ponzio 34/5, 20133 Milano, Italy; 2 BiomimX S.r.l., Via G. Durando 38/A, 20158 Milano, Italy

**Keywords:** Mechanical stimulation, Cardiac microtissues, Maturation

## Abstract

The most advanced in vitro cardiac models are today based on the use of induced pluripotent stem cells (iPSCs); however, the maturation of cardiomyocytes (CMs) has not yet been fully achieved. Therefore, there is a rising need to move towards models capable of promoting an adult-like cardiomyocytes phenotype. Many strategies have been applied such as co-culture of cardiomyocytes, with fibroblasts and endothelial cells, or conditioning them through biochemical factors and physical stimulations. Here, we focus on mechanical stimulation as it aims to mimic the different mechanical forces that heart receives during its development and the post-natal period. We describe the current strategies and the mechanical properties necessary to promote a positive response in cardiac tissues from different cell sources, distinguishing between passive stimulation, which includes stiffness, topography and static stress and active stimulation, encompassing cyclic strain, compression or perfusion. We also highlight how mechanical stimulation is applied in disease modelling.

## Introduction

**Fig. 1:**
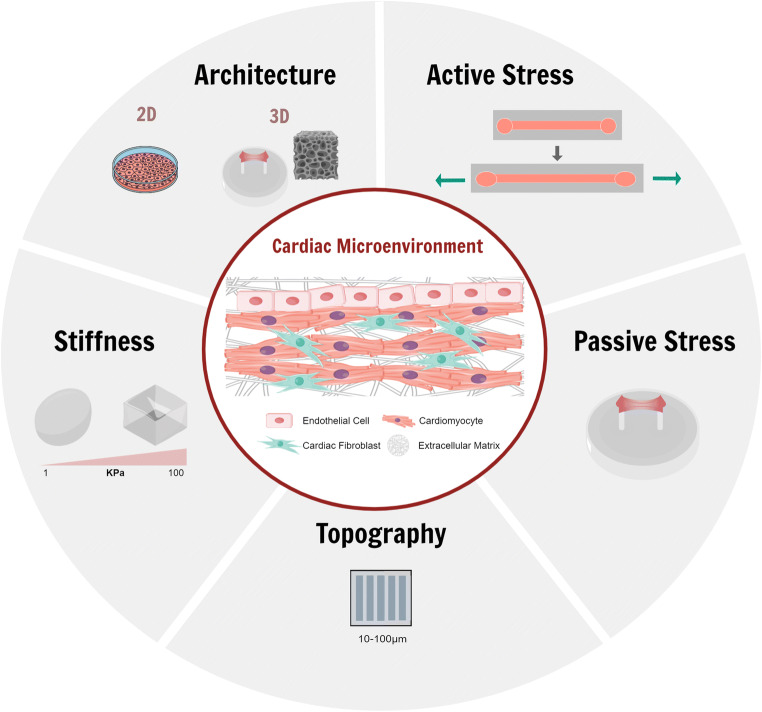
Cardiac microenvironment and mechanical stimuli applied aimed to mimic adult cardiac microtissue

The current animal models, used in drug development, do not fully recapitulate the structure and physiology of the human heart (e.g. heart rate of mouse is 10 times faster than in humans), failing to mimic both cardiac human healthy or disease conditions. Recently, to overcome this issue, in vitro models have gained more interest in the scientific community. Specifically, the most advanced in vitro cardiac models are today based on the use of induced pluripotent stem cells (iPSCs), which open the path to the modelling of person-specific characteristics, either in systems that use cell aggregates (e.g. spheroids, organoids), scaffold-based techniques or organ-on-chip approaches. However, full recapitulation has not yet been fully achieved in either of the systems mentioned, urgently raising the need to move towards models capable of promoting an adult-like cardiomyocyte phenotype. Currently, the maturation of cardiomyocytes (CMs) is determined by improvements on genetic cardiac markers and key functional parameters such as electrical propagation or contraction force on the engineered cardiac tissues. Some of the proposed methods to potentiate CM maturation rely on their co-culture with other cardiac cell types (e.g. endothelial cells, cardiac fibroblasts), long-term culture, electric and/or mechanical stimulation and alteration of the biochemical environment (Guo and William 2020; Karbassi et al. 2020). Mechanical stimulation has been considered for cardiac maturation with the aim to mimic the different mechanical forces that the cardiac tissue undergoes over the course of development. For instance, (1) shear stress, generated after tube formation due to blood flow; (2) cyclic strain, affecting cardiomyocytes after the first heart beat according with the systolic and diastolic rhythm; (3) hydrostatic stretching initiated after birth due to increased blood pressure (Hendrickson et al. 2021; Liao et al. 2021) and (4) forces derived from the elastic modulus of ECM, increasing from neonatal tissue (< 10 kPa) to adult heart tissue (15–30 kPa) (Gaetani et al. 2020; Guo and William 2020; Stoppel, Kaplan, and Black 2016). In this review, we provide an overview of the current strategies applied to mechanically stimulate cardiac microtissues that are summarized in Table 1. We first describe the passive stimulation that includes changing stiffness, topography and passive strain, then we explain the current methods for active stimulation and its effects on cardiac maturation and finally, we give a perspective of how mechanical stimulation is used in disease modelling (Fig. 1).

**Table 1:** Summary of different mechanical stimulations used to engineer cardiac microtissues

**Publication**	**Cell type**	**Mechanical stress**	**Substrate**	**Conditions**	**Time of duration**	**Other stimulations**
**Rodriguez et al. (**2019**)**	hESC-CMs	Stiffness	PDMS	5–101 kPa at 12–24 μm width	9 days	
**Jacot et al. (**2008**)**	NRVM	Stiffness	Collagen coated polyacrylamide gel	1–50 kPa	7 days	
**Lu et al. (**2021**)**	hiPSC-CM	Passive	Collagen hydrogel	0–0.32 mm/day	21 days	Electrical stimulation: 1 Hz and 50 mA
**Lui et al. (**2021**)**	hiPSC-CM	Passive	Scaffold-free	12.5% uniaxial stretch	Up to 4 weeks	
**Ruan et al. (**2016**)**	hiPSC-CM	Passive	Collagen based		2 weeks	Electrical stimulation: 2nd week at 2 Hz, 5V/cm, 5 ms pulse
**Hinson et al. (**2015**)**	hiPSC-CM	Passive	Collagen based	0.2–0.45 μN/μm cantilevers	4 days	Electrical stimulation: biphasic square pulses 1ms, 1Hz
**Rogers et al. (**2019**)**	RFB	Cyclic stretch and compression	Fibrin	Compression: 1 Hz, 10–160 mmHg Strain: 0–7% 1 Hz	7 days	
**Parsa et al. (**2017**)**	NRCM	Cyclic stretch	Collagen	5 Hz 0–10% 1:2 duty cycle	7 days	
**Tulloch et al. (**2011**)**	hiPSC-CM/+HUVEC/+MSC/+MEFs NRCM	Static or cyclic stretch	Collagen I	1 Hz, 5% elongation	4 days	
**Kreutzer et al. (**2020**)**	hiPSC-CM	Cyclic stretch	PDMS with gelatine	0.5–0.8Hz at 1.5–8% elongation	Up to 10 days	
**Cortes et al. (**2020**)**	hiPSC-CM	Cyclic stretch	Silicon membrane	1.33 Hz, 8.9–0.02%	Up to 7 days	Electrical stimulation: 5V/cm, 5 ms 10% duty cycle (current density 13 mA cm^−2^)
**Massai et al. (**2020**)**	NRCM	Cyclic stretch	Fibrin	1 Hz 10%	4 days	Electrical stimulation for functional analysis: 1 Hz, 1V/cm, 2 ms square pulse
**McCain et al. (**2013**)**	NRCM	Cyclic stretch	PDMS	3 Hz 10%	4 days	Electrical stimulation for functional analysis: 2–5 V 2 Hz
**Marsano et al. (**2016**)**	hiPSC-CM NRCM	Cyclic stretch	Fibrin	1 Hz 10%	5 days	Electrical stimulation for functional analysis: After mechanical maturation (day 5), 1–10 Hz, 0–23 V, 4 ms square pulse
**Shachar et al. (**2012**)**	NRCM​	Compression Shear stress	Alginate scaffold	Compression: 1 Hz 15% Shear stress: 10^−2^–10^−1^ dynes/cm^2^	Intermittent: 4 days, 30’ daily Continuously: 4 days	
**Cruz-Moreira et al. (**2021**)**	NRCM	Shear stress	Fibrin	1.4–2.16 dynes/cm^2^	5 days	Electrical stimulation:1-10Hz, 0-10V, 4ms monophasic.
**Visone et al. (**2018**)**	NRCM	Cyclic stretch	Fibrin	1 Hz 10%	5 days	Electrical stimulation: 0.01 Hz–1 kHz, ± 0.001–10 V monobiphasic square 0.4% duty cycle
**Liaw and Zimmerman et al. (2002)**	NRCM	Cyclic stretch	Matrigel	2 Hz 10%	7 days

## Passive stimulation

Passive mechanical stimulation can be obtained by either modulating the stiffness (Jacot, McCulloch, and Omens 2008; Rodriguez et al. 2019) and the topography (Lind et al. 2017; Rodriguez et al. 2019) of the substrate or providing static stresses to 3D cardiac microtissues (Lu et al. 2021; Lui et al. 2021; Ruan et al. 2016). These methods have been integrated in advanced cell culture systems and proved to be easily tuneable based on the specific objectives, such as modelling physiological states for drug screening or inducing pathological characteristics for disease modelling.

**Fig. 2:**
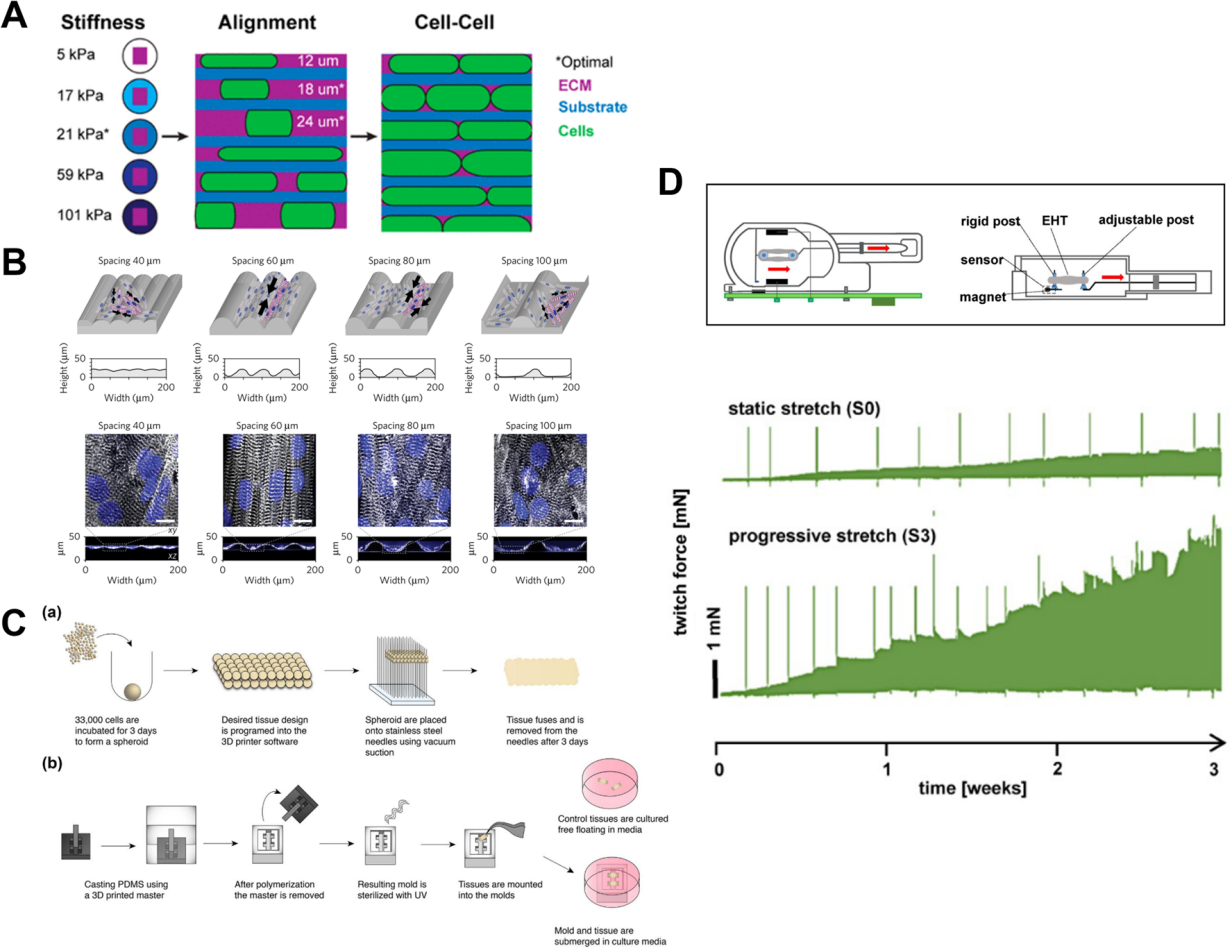
Overview of different studies exploiting passive stimulation. A hESC-CMs were seeded onto PA substrates to study the effect of stiffness, cell anisotropy and cell-cell contact on cardiac maturation. (Rodriguez et al. 2019). B Microgrooves obtained through 3D printing were used to study the influence of surface topography on cell anisotropic re-arrangement. (Lind et al. 2017). C A scaffold-free approach with tri-culture (cardiomyocytes, fibroblasts and endothelial cells) was developed to study how passive stretch affects cardiac microtissue without the confounding effect of the biomaterial support. (Lui et al. 2021). D Progressive stretch (S3) over 3 weeks was compared to static stretch (S0) showing increased twitch force (Lu et al. 2021)

Substrate stiffness has started to be considered as a key stimulation component not only to mimic the physiological development of the heart but also its pathological states as well as aging processes. In vitro, an elastic modulus of 10 kPa showed to promote cell alignment, larger calcium transients and higher sarcoplasmic calcium storage within neonatal rat cardiac cells cultured in 2D on polyacrylamide (PA) gel, as compared to 1 kPa, 5 kPa, 25 kPa and 50 kPa elastic modulus (Jacot et al. 2008). In a more recent study, cardiomyocytes derived from human embryonic stem cells (hESC-CMs) were cultured, as a monolayer, onto polydimethylsiloxane (PDMS) substrates exhibiting elastic moduli ranging from 5 to 101 kPa (Fig. 2A). An increased cell spread area, binucleation and maximal calcium intensities were observed in cells plated on surfaces with 21 kPa stiffness as compared to other substrates (Rodriguez et al. 2019). While clearly demonstrating that cardiomyocytes selectively respond to stiffness signalling, the same work showed that a combination with other stimuli might lead to closer adult-like state. For instance, substrate topography also plays a role in cardiomyocyte maturation by improving the anisotropic state of cardiac tissue and facilitating the electrical signal propagation. To demonstrate this concept, microcontact printing was used to pattern laminin on lines with widths of 12, 18 and 24 μm on PDMS substrates. hEPSC-CMs cultured in 2D on 18 and 24 μm wide patterns showed superior structural and functional properties as compared to thinner structures, as well as an increased cell aspect ratio (less elongated) and a decreased cell area. Moreover, in the same work, the authors tested higher cell density, to assess the contribution of cell-cell interactions: the higher density (~ 1000 cells/mm^2^) increased cell area, cell aspect ratio, sarcomere length, binucleation and faster calcium transient as compared to the lower density (~ 500 cells/mm^2^). These findings showed that the integration of stiffness, anisotropy and cell-cell contact are of paramount importance for improving cardiac structural and functional properties (Rodriguez et al. 2019). Besides contact printing, an additional promising technique used to design substrate topography is 3D printing. The group of Parker (Lind et al. 2017) developed the so-called “cardiopatch” system, which is provided with controlled micro-grooves obtained by printing filaments using shear-thinning soft PDMS as ink. A spacing between grooves of 60 μm promoted neonatal rat ventricular myocyte (NRVM) alignment and developed 2D anisotropic engineered tissues with the highest longitudinal to transverse velocity ratio of action potential propagation as compared to 40, 80 and 100 μm grooves (Fig. 2B).

In order to obtain more complex and biologically reliable models, mechanical stimulation has also been applied in 3D in vitro models. For instance, static stress conditioning is usually achieved by maintaining cardiac constructs at a fixed static length; over time, cell sarcomeres rearrange and the internal stress increases together with the construct maturation. Ruan et al. (2016) applied such static stress by maintaining microtissues of cardiomyocytes derived from human-induced pluripotent stem cells (hiPSC-CMs) at a fixed static length for 2 weeks between pairs of nylon tabs. Such static stress promoted cell alignment, cardiomyocyte hypertrophy, increased contractility and passive stiffness and improved cell force-frequency relationships. To study the effect of progressive stretch on hiPSC-CMs derived cardiac tissues, K.Lu and colleagues used biomimetic cultivation chamber that allows the control of preload, elastic systolic load, electrical stimulation and medium agitation (Fig. 1D). After 21 days in culture, the cardiac tissues conditioned with the highest stretch (i.e. 0.32 mm/day) showed an increased contractility (i.e. 11.3 mN/mm^2^), sarcomere length (i.e. 2.2 μm) and passive stiffness (i.e. 4.5 kPa), as compared to cardiac tissues in static condition (static length) or subjected to a progressive stretch of 0.08 and 0.16 mm/day. Of note, these achievements are not only consequences of the progressive stretch, but are also related to the combination of electrical stimulation and enhanced oxygen supply thanks to an integrated rocker mixer (Lu et al. 2021).

In 3D engineered heart models, most of the cardiac tissues are generated from cardiac cells embedded within a scaffold, usually a hydrogel, mainly based on fibrin or collagen (Zuppinger 2019). While this approach mimics the 3D architecture of the cardiac tissue, the inappropriate tuning of the hydrogel mechanical properties may lead to undesirable cell-biomaterial interactions, which, in turn, often lead to unexpected results, where the contribution of the stretching on model maturation cannot be properly discerned. To avoid the confounding effect derived from supporting biomaterials, C. Lui exploited a scaffold-free approach relying on self-assembling spheroid formation, where cardiomyocytes and supportive cells, i.e. 15% of cardiac fibroblasts and 15% of endothelial cells (HUVECS) produced their own ECM (Fig. 2C). By applying to the model a 12.5% uniaxial static stretch for 1 and 4 weeks, the sarcomere length increased (1.51 ± 0.31 μm and 1.90 ± 0.12 μm after 1 and 4 weeks, respectively), the elastic modulus of the aggregate decreased, and known cardiac markers such as MYH7, CASQ2 and SERCA2 were upregulated. In addition, after stretching, the alignment of collagen I, collagen III and vimentin fibrils increased, evidenced by the fibroblast capacity of reorganization (Fig. 2C) (Lui et al. 2021). Fibroblasts have been proposed in several models to respond to mechanical stimulation, potentially interfering with cardiomyocyte maturation, making this aspect especially relevant for co-culture approaches (Giacomelli et al. 2020). The excessive activation of fibroblasts and their transition into myofibroblasts in response to extreme mechanical load, for instance, have been shown to increase ECM deposition and stiffness. This may cause a loss in cardiomyocyte functionality, resulting in cardiac fibrosis (Mainardi et al. 2021). The activation of myofibroblasts has been shown to be attenuated at physiological strains (5–10%) while it is augmented at pathological ones (10–20%) (Kong et al. 2019).

The strategies presented above highlight the improvements achieved in modelling the cardiac tissue, either by exploiting surface stiffness, topography, passive or stepwise stretch. Moreover, the relevance of scaffold-free approaches and the implementation of other cardiac cell types in addition to cardiomyocytes, such as fibroblasts, were demonstrated to be highly relevant to fully explore the cellular response to mechanical load. Although the progressive stretch was shown to lead to great improvements in driving the maturation of cardiomyocytes, a major drawback is represented by the limit in the tissue displacement that can cause tissue disruption during mechanical deformation (Lu et al. 2021).

## Active stimulation

**Fig. 3:**
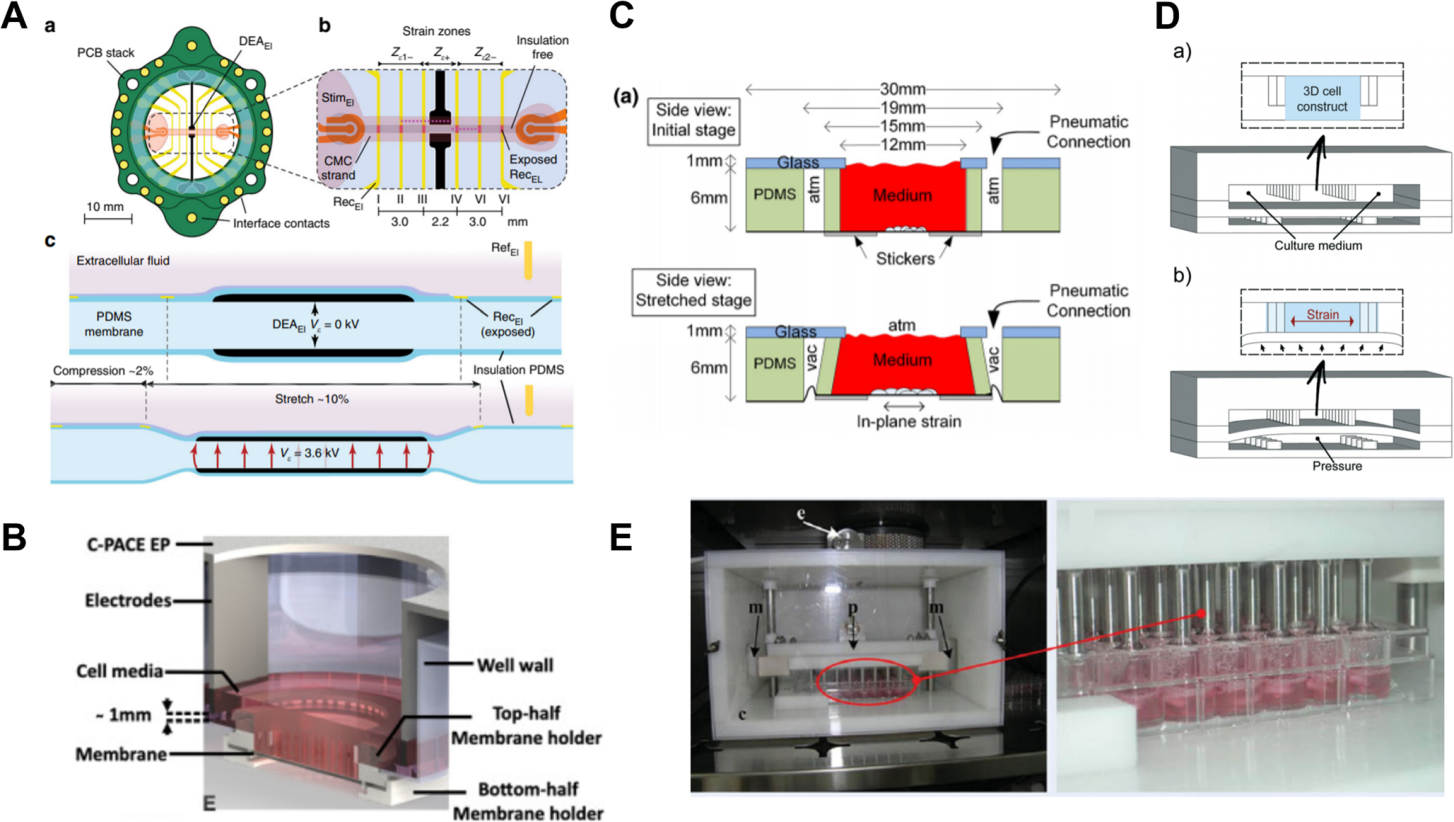
Overview of studies using active mechanical stimulation. A The so called mechano-active multielectrode-array, MaMEA allows the compression and stretch of cardiac microtissue integrated with electrodes (Imboden et al. 2019). B “BeatS-α” a 3D printed device capable of mechanical and electrical stimulation (Cortes et al. 2020). C Uniaxial stimulation was used to stimulate hiPSC-CMs (Kreutzer et al. 2020). D The beating-heart-on-chip platform cable to provide mechanical stimulus through a pneumatic platform (Marsano et al. 2016). E Cyclic compression was applied and compared with steady-state compression (Shachar et al. 2012)

In the last decade, cardiac tissue engineering has taken advantage from advanced culture platforms able to provide cardiac cells with dynamic mechanical stimulations (Liaw and Zimmermann 2016; Stoppel et al. 2016). Among others, uniaxial strain is often used to stretch cell constructs in the attempt to recapitulate the complex mechanical environment experienced by cardiovascular cells in vivo (Gupta and Jane Grande-Allen 2006). For instance, a platform (mechano-active multielectrode-array, MaMEA) was developed to apply uniaxial stretch and compressions while making electrophysiological measurements through dielectric elastomer actuators. In detail, upon the application of an electric field, the actuators, made of a smart material called dielectric elastomer, caused the deformation of a PDMS membrane which in turn transfers a uniaxial stretch and compression in defined regions of the substrate, where 2D NRVM strands are generated. The electrodes positioned across the biological samples allowed for measurement of electrical signal propagation during the stimulation (Fig. 3A) (Imboden et al. 2019). A similar device has been also developed to mechanically stimulate cardiac fibroblast monolayers through lateral pneumatic actuators stretching a PDMS anchored membrane (Ugolini et al. 2016). A 2–8% cyclic strain applied for 1–3 days increased cellular proliferation and elongation and was demonstrated to be sufficient to induce the reallocation of YAP, a mechanosensing-associated transcription factor modulating the proliferative cell response (Dupont et al. 2011), from cytoplasm to the nucleus. In a different study, 2D cells were subjected to electromechanical stimulation by integration of flexible membrane-based actuators via 3D printing: Cortes and collaborators demonstrated that iPSC-CM improved their maturation with increased expression ratio of cardiac troponin isoforms (TNNI3) and lower skeletal expression (TNNI1) TN+ after 7 days of electromechanical stimulation (Fig. 3B) (Cortes et al. 2020).

The active mechanical stimulation has also been applied in cardiac differentiation studies to enhance maturation of the in vitro models. Kreutzer et al. developed a circular plate coupled with an enclosed vacuum chamber ring (“BeatS-α”). When negative pressure was applied, the vacuum ring displaced a membrane onto which cells were cultured in monolayer, thus providing them with a mechanical stretch (Fig. 3C). The system promoted the cardiac differentiation from different human PSCs using 5% cyclic strain, as confirmed by the increased expression of cardiac genes (i.e. MYH6, MYH7, Cx43, TropI) (Kreutzer et al. 2014). More recently, the same platform was exploited to culture hiPSC-CM at 8% cyclic strain, obtaining a sarcomere alignment perpendicular to the strain direction (Kreutzer et al. 2020). Similar results on sarcomere alignment for hiPSC-CM maturation were obtained by Wenkon Dou et al. using cardiac monolayers (Dou et al. 2021). The authors used a pneumatic stretchable platform applying different mechanical loads (5%, 10%, 15% and 20% of strain magnitudes at 1Hz) from days 2 to 10, from beneath the tissue. The magnitudes 15% and 20% resulted in 9.4% increased values of sarcomere length, upregulation of the MYH7 expression in a strain-dependent manner and improved contractility up to 15%.

In the attempt to develop more relevant 3D cardiac models, 3D mechanically active systems have been engineered (Stoppel et al. 2016). Marsano et al. developed an innovative beating heart-on-chip platform able to provide homogeneous cyclic uniaxial mechanical strain to cardiac microtissues by using a PDMS membrane interposed between the culture chamber and an actuation compartment (Fig. 3D). The 3D biological construct was generated between two rows of hanging posts, which were used to limit the membrane displacement, allowing the accurate control of the strain level sensed by the cells (Marsano et al. 2016). The mechanical training (i.e. 10% uniaxial strain at 1Hz) provided to either NRVM or hiPSC-CM based microtissues was shown to promote the maturation of the constructs, which showed spontaneous synchronous beating and enhanced contractile capabilities, such as a lower excitation threshold, and a higher amplitude and contraction velocity. The same mechanical stimulation has already been further exploited for disease modelling, demonstrating the ability of the platform to reproduce in vitro some of the key steps of cardiac fibrosis (Occhetta et al. 2018). More recently, the integration of electric systems allows for stimulation and sensing capabilities of the beating heart-on-chip simultaneously. The potential benefits of applying either a mechanical, an electrical or a combined electro-mechanical stimulation, to promote the contractile capabilities of cardiac microtissues, were corroborated (Visone et al. 2018). Moreover, the incorporation of sensing electrodes to record online the field potential generated by cardiac microtissues demonstrated how the mechanical stimulation gradually improves the electrical maturation of the constructs, which showed an increased synchronicity and a lower variation of the beating frequency after 5 days in dynamic culture conditions. The system was also demonstrated suitable to perform drug cardiotoxicity screening, by monitoring the electric field alteration caused by compounds in both murine and hiPSC-CM with human fibroblasts (Visone et al. 2021).

Different macroscale systems, such as custom-made bioreactors incorporating different stretching apparatus, have also been developed to actively subject engineered heart tissues (EHTs) to mechanical stimulation (Kensah et al. 2011; Mihic et al. 2014; Ruan et al. 2015). For all, beneficial effects have been shown in terms of tissue maturation and cellular alignment. In order to develop a platform with active cyclic stress, able to accompany the maturation stage, Massai and colleagues developed ring-shaped 3D NRVM constructs and subjected them to a cyclic stretch of 10% strain at 1 Hz for 4 days. From the comparison with static conditions, the mechanical stimulus increased alignment, maturation markers and force of the developed cardiac constructs, achieving an improved cardiac contractile activity, with more synchronous and regular contractions in mechanically trained constructs (Massai et al. 2020).

Fewer examples of other types of stress, such as compression and shear stresses, have also been reported. Sachar et al. (Fig. 3E) demonstrated the differences between continuous or intermittent (i.e. 30 min each day) cyclic compression provided in combination with fixed shear stresses (10^−2^–10^−1^ dynes/cm^2^) in 3D constructs made of rat cardiomyocytes (Shachar, Benishti, and Cohen 2012). Intermittent cyclic compression yielded to a more aligned cellular morphology, compared to circular cell shape in continuously compressed tissues and better preserved key cardiomyocyte markers (α-actinin and n-cadherin) as compared to non-stimulated constructs. Other recent studies demonstrated the key role of physiological level of flow in improving functional performance and enhancing cellular alignment of both 2D and 3D systems (Cruz-Moreira et al. 2021; Kobuszewska et al. 2017).

As seen before, co-culture strategies promote cardiomyocyte maturation (Giacomelli et al. 2020) and, as such, it is important to characterize the effect of mechanical load on other cardiac cells then CMs. Tulloch et al. cultured hESC-CMs and iPSC-CMs within 3D collagen matrix and subject them to either static stress or cyclic stress-conditioning (i.e. 1Hz at 5% of elongation) for 4 days (Tulloch et al. 2011). Static stress enhanced myofibrillogenesis and sarcomeric bands, while both mechanical stress regimens promoted cellular alignment and increased proliferation and hypertrophy markers. Moreover, the same mechanically trained cardiac tissues provided with endothelial cells developed internal vessel-like structures and showed an improved cardiomyocyte proliferative activity.

## Pathology and disease models

**Fig. 4:**
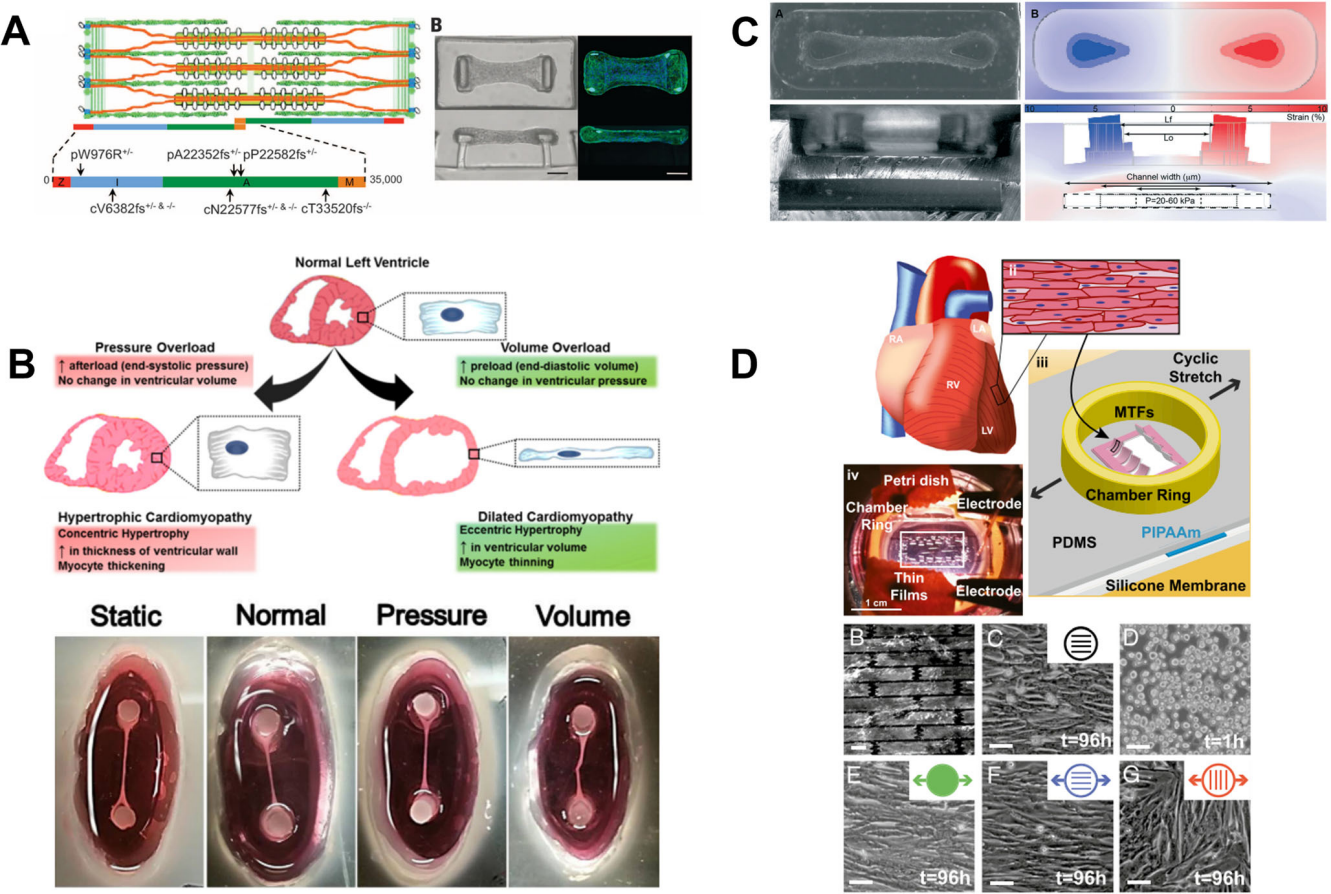
Overview of different studies exploiting mechanical stimulation for disease modelling. A Rigid cantilevers (0.45 mN/mm) were seen to impair cardiomyocytes with titin mutation (Hinson et al. 2015). B Pressure and volume load were used to mimic hypertrophic and dilated cardiomyopathy (Rogers et al. 2019). C Cyclic stress was applied, and cardiac hypertrophy was shown as a result of the mechanical stimulus (Parsa et al. 2017). D 10% cyclic stress at 2–3Hz was used to mimic the failing myocardium (McCain et al. 2013)

As cardiac models progress, the pathological heart features have been increasingly examined to elucidate the specific molecular mechanisms leading to different cardiac disease states with the final aim to find new treatments (Brandao et al. 2017). In cardiac pathologies, the mechanical stimulation has been recognized to play an important role (Mills et al. 2018). Recently, the effect of stiffness on Duchenne muscular dystrophy (DMD) cardiomyopathy was uncovered using hiPSCs derived from DMD patients, healthy individuals and isogenic controls (i.e. DMD mutation corrected or introduced). Using a tuneable hydrogel and traction force microscopy, DMD cardiomyocytes were demonstrated unable to compensate for dystrophin deficiency when cultured on substrates with fibrotic-like elastic modulus (i.e. 35 kPa), showing impaired functionality while maintaining the features of DMD cardiomyopathy on 10 kPa hydrogels (Chang et al. 2021). Different approaches aiming at understanding the triggers and mechanisms of cardiac pathologies rely on application of non-physiological stimuli to healthy cardiomyocytes. For instance, increased afterload was seen to provoke cardiomyocyte hypertrophy accompanied by the activation of the foetal gene program (e.g. atria natriuretic peptide (ANP), β-myosin heavy chain (MYH7) and skeletal α-actin (ACTA)) and led to fibrotic activation and impairment of contractile force and relaxation in fibrin-based EHT casted between silicone posts (Hirt et al. 2012). This detrimental phenomenon could be prevented by administration of endothelin blockers for decreasing hypertrophy, suggesting the exploitation of the model to find new effective treatments. Pathological remodelling was also introduced through cyclic stretch at 10% strain at 3 Hz frequency in a laminar ventricular tissue on a chip, which showed a specific decrease of α-to β-myosin heavy chain ratios, anomalous myocyte shape and sarcomere misalignment (Fig. 4D) (McCain et al. 2013). Pursuing cardiac hypertrophy modelling, Parsa and colleagues created a microfluidic platform where rat cardiomyocytes were cultured in 3D collagen gels constricted between two pillars and subjected to a 5-Hz stretch with variable strain (i.e. 0%, 5% and 15%) to mimic volume overload (Fig. 4C) (Parsa, Wang, and Vunjak-Novakovic 2017). Through this platform, tests were performed in a high-throughput manner: by tuning the pneumatic loading of microtissues, the hypertrophic response was shown to correlate to the strain in a dose-dependent manner.

Mechanical stimulation has shown great progresses and has increasing significance, especially in disease modelling (Mills et al. 2018). However, the impairment of pressure/volume load process, which is the base of many cardiac pathologies, was poorly considered (Fig. 4B) (Rogers et al. 2019). In this context, cardiac tissue chips (CTCs) were developed to mimic either pressure overload (i.e. 1 Hz, 160 mmHg peak-systolic pressure, 10 mmHg end-diastolic pressure and 0–2% strain) or volume overload (i.e. 1 Hz ,100 mmHg peak-systolic pressure, 30 mmHg end-diastolic pressure, and 2–7% strain) to reproduce hypertrophic cardiomyopathy and dilated cardiomyopathy, respectively (Rogers et al. 2019). The study of gene expression of fibrosis-related proteins in H9c2 cell line derived from embryonic BD1X rat heart evidenced that the pressure overload upregulated collagen I and TGF-β, inducing hypertrophic remodelling and fibrosis, while the volume overload led to an overexpression of desmin, enhancing structural support and obtaining a thin and more elongated engineered tissue.

Mechanical stimulation becomes especially relevant when using fibroblasts in co-culture with cardiomyocytes. Kong and colleagues studied the effect of mechanical stimulation in the activation and phenotypic transition from cardiac fibroblasts (CFs) to myofibroblasts. By varying the compression ranges, it was evidence that that physiological strains (i.e. 5–10%) can maintain or attenuate myofibroblast transition, while pathological strains (i.e. 10–15%) promote activation of myofibroblasts, inducing traits of cardiac fibrosis (Kong et al. 2019).

Patient-derived iPSCs also bring possibilities to study disease mechanisms linked to inherited mutations (Bellin and Mummery 2016). Titin or BRAF mutations in iPSC-CMs were already used to model phenotypes characteristic of dilated cardiomyopathy and hypertrophic cardiomyopathy, respectively (Cashman et al. 2016; Hinson et al. 2015). The effect of mechanical stress on titin mutated cells was investigated by comparing microtissues grown around flexible (0.2 mN/mm) and rigid (0.45 mN/mm) cantilevers and revealed their impaired cardiac adaptation to increased mechanical load (Fig. 4A) (Hinson et al. 2015).

As described, non-physiological mechanical stimuli have been extensively exploited to model pathological states of the cardiac tissues, offering also innovative tools to specifically reproduce intrinsic characteristics of different diseases and to understand their main causes.

## Conclusions and perspective

Over the last two decades, the scientific community has made relevant advances in mimicking adult cardiac microtissues. The availability of new technologies such as iPSC, 3D-printing and microfluidics, together with previous knowledge on 2D models and the tunability of biomaterials, has allowed the generation of sophisticated systems and mechanisms capable of increasing tissue maturation towards levels close to the adult human heart, both in terms of morphology (e.g. sarcomere length), molecular (e.g. increased adult-like gene expression) and functional (active forces and contractile stresses) (Ronaldson-Bouchard et al. 2018; Shadrin et al. 2017). Mechanical stimulation represents an essential ingredient to enhance the maturation of cardiac in vitro models, which is pivotal to improve the drug screening process or the discovery of new treatments for heart complications, as well as to develop models of cardiac diseases. Simple, yet effective methods for the mechanically conditioning of cardiomyocytes can be referred to as passive stimulation, in which no active force fields are used. Examples include the control of substrate stiffness, topography and passive strains. Although effective to achieve a higher in vivo-like organization and functionality of 2D cardiac models and an improved cell maturation in 3D cardiac tissues, the systolic and diastolic rhythm is not considered in these systems. Indeed, more advanced solutions envisage active stimulations, which allow cyclic loading/deformations or even more complex time-varying patterns to better recapitulate the mechanical environment of the heart. Examples include pneumatic active membranes that stretch biological cardiac constructs providing synchronous beating (Marsano et al. 2016), dielectric actuators (Imboden et al. 2019) that provide uniaxial mechanical strain or even mechanical compression for enhanced morphology (Shachar et al. 2012). Of note, to assess the maturation of cardiac models, methods to sense tissue properties have been developed: most systems able to perform a mechanical characterization rely on end-point analysis. However, technological solutions to perform online measurements are gaining interest, and such systems promise to quantify cell contractility changes over time during maturation, either through optical mapping or flexible micropillars (Dostanić et al. 2020; Dou et al. 2021; van Meer et al. 2019). The addition of these new features in static or dynamic stimulation systems, combined with tunable stiffness and topography, is expected to dramatically contribute to achieve reliable cardiac models to study physiological and pathological mechanisms in the near future.
