# Correction to: Current strategies of mechanical stimulation for maturation of cardiac microtissues

**DOI:** 10.1007/s12551-022-00940-y

**Published:** 2022-03-21

**Authors:** Maria Carlos-Oliveira, Ferran Lozano-Juan, Paola Occhetta, Roberta Visone, Marco Rasponi

**Affiliations:** 1 Department of Electronics, Information and Bioengineering, Politecnico di Milano, Via Ponzio 34/5, 20133 Milano, Italy; 2 BiomimX S.r.l., Via G. Durando 38/A, 20158 Milano, Italy

**Correction to: Biophysical Reviews (2021) 13:717–727**

**https://doi.org/10.1007/s12551-021-00841-6**

In the original version of the article, the second sentence in the Funding section needs to be modified. The correct section is given below:

## Funding

Open access funding provided by Politecnico di Milano within the CRUI-CARE Agreement. This project has received funding from the European Union’s Horizon 2020 research and innovation programme under the Marie Skłodowska-Curie grant agreement No 860715. This project has received funding from the European Union’s Horizon 2020 research and innovation programme under the Marie Skłodowska-Curie grant agreement No 841975.

